# Defect-Driven Neuromorphic Plasticity in Planar ZnO Optoelectronic Synapses

**DOI:** 10.1021/acsami.5c21253

**Published:** 2026-01-13

**Authors:** Zhiyuan Ren, Shan Wang, Bingheng Meng, Huan Liu, Qing An, Longxing Su, Rui Chen

**Affiliations:** 1 Department of Electrical and Electronic Engineering, 255310 Southern University of Science and Technology, Shenzhen, Guangdong 518055, P. R. China; 2 International School of Microelectronics, 74549 Dongguan University of Technology Dongguan, Guangdong 523808, P. R. China; 3 Institute of Applied Physics and Materials Engineering, University of Macau Avenida da Universidade, Taipa, Macao 999078, P. R. China

**Keywords:** optoelectronic synapse, wide-band gap semiconductor, defect, carrier dynamics, plasticity

## Abstract

Understanding how atomic-scale defect dynamics influence system-level neuromorphic behavior is crucial for the rational design of oxide-based optoelectronic synapses. In this study, a planar ZnO synapse has been introduced where the nanosecond-scale oxygen-vacancy carrier lifetime is directly linked to second-scale persistent photoconductivity (PPC) decay and key synaptic plasticity parameters. By combining steady-state and time-resolved spectroscopies with electrical measurements, a dynamic framework that spans multiple time scales has been developed: long-lived defect states slow PPC decay, which in turn regulates paired-pulse facilitation retention and the efficiency of short-to-long-term plasticity transitions. This framework allows for predictive tuning of the synaptic weight by controlling defect occupation and release kinetics. The optimized ZnO synapse operating at 0.1 V demonstrates robust long-term potentiation and achieves 90.8% recognition accuracy in handwritten digit recognition. This work presents a cross-time scale design strategy that bridges atomic-level defect engineering with neuromorphic system performance, paving a route toward artificial vision hardware.

## Graphical abstract

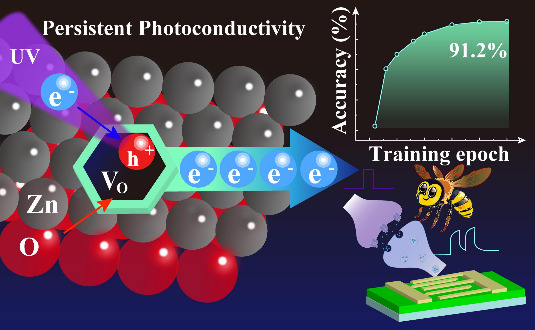

## Introduction

Artificial intelligence requires a revolutionary computing architecture. The von Neumann bottleneck, which arises from the physical separation between memory and processing units, limits computational speed and efficiency.^1–3^ Neuromorphic computing, which mimics the structure and function of biological neural networks, provides a promising solution to this challenge. In the brain, synaptic plasticity enables efficient information processing through dynamic neural connections. Optoelectronic synapses, which use light for signaling, have emerged as a highly promising neuromorphic component,^4,5^ significantly reducing electrothermal losses. Their nonvolatile photoresponse emulates biological plasticity, offering distinct advantages for high-speed systems.^6–8^ Effective optoelectronic synapses must integrate sensing, memory, and processing with low energy consumption. Drawing inspiration from human vision, which processes approximately 80% of external information,^9^ these devices play a critical role in the development of artificial visual systems.^10^

Current research focuses on various material systems,^11–13^ including oxide semiconductors (e.g., ZnO, TiO_2_), perovskites (e.g., MAPbI_3_), and 2D materials (e.g., MoS_2_).^3,14–20^ These materials are typically employed in one of three device structures, namely memristors, two-terminal structures, and three-terminal transistors.^21,22^ Among them, two-terminal devices based on wide-band gap oxide semiconductors are of particular interest due to their tunable persistent photoconductivity (PPC) effect. Consequently, controlling the duration of the PPC effect has become a key research focus. Kim et al. achieved a 10^4^-second PPC in In_2_O_3_ films by controlling the trapping of oxygen vacancy (V_O_),^23^ while Liu’s team suppressed the PPC in Ga_2_O_3_ devices through junction engineering to improve high-gain photodetection.^24,25^ Alternative strategies have been explored to modulate the PPC lifetimes. For instance, Ajjel et al. used Au nanoparticles to modify TiO_2_ interfaces to achieve programmability of the PPC lifetime by modulating the carrier trapping energy level.^26^ These studies have clarified the critical role of PPC in regulating synaptic weights across the oxide semiconductor family. Among oxide semiconductors, ZnO exhibits unique potential with multifunctional features in transparent conductive films, electronic devices, and optoelectronic devices. As an environmentally friendly and abundant wide-band gap semiconductor, the concentration of V_O_ defects can be well controlled during the preparation process, making it an ideal platform for studying defect-driven PPC. Furthermore, its strong photoresponse in the ultraviolet (UV) region aligns well with bioinspired concepts. For example, bees use UV vision for navigation and foraging, while reindeer rely on UV vision to search for lichens. Indeed, the PPC dynamics in ZnO has been receiving considerable attention during the past few years. For example, Yu’s team employed variable-temperature photocurrent analysis on ZnO nanowires, revealing that the PPC dynamics are dominated by the thermal activation of shallowly trapped electrons.^27^ However, a comprehensive understanding of the spatiotemporal dynamics of oxygen vacancies in ZnO semiconductors, particularly their direct impact on synaptic function, still remains to be thoroughly investigated.

In this work, a two-terminal optoelectronic synapse based on a ZnO thin film is fabricated, and its synaptic functions are systematically investigated. Various optical spectroscopies, such as photoluminescence (PL) and time-resolved PL (TRPL) measurements, have been performed to quantify the carrier dynamics. Various synaptic functions have been characterized, including paired-pulse facilitation (PPF), frequency-dependent learning, and the critical transition from short-term plasticity (STP) to long-term potentiation (LTP). The results reveal that the slow relaxation of charge carriers trapped in V_O_ defect states is the primary mechanism controlling the device’s learning and memory processes. The study establishes a direct correlation between microscopic carrier lifetimes and macroscopic synaptic behaviors, providing a predictive design framework for neuromorphic devices.

## Results and Discussion

Figure 1 illustrates the bioinspired design philosophy of an optoelectronic synapse based on ZnO thin film. The biological neuron shown schematically in Figure 1a serves as the functional model, which contains a pre-synapse and a post-synapse. The excitatory post-synaptic current is induced by the external spikes in the pre-synapse, which can be imitated with a honeybee’s visual system, including an external UV stimulation (Input) and a post-synaptic response (Output). To physically realize this concept, we fabricated the planar artificial synaptic device shown in Figure 1b. Its architecture directly mirrors the biological system: two pairs of coplanar Au/Cr electrodes function as the pre- and post-synaptic terminals, while the ZnO semiconductor channel acts as the artificial synaptic cleft. The honeybee highlights the inspiration drawn from insect visual systems, whose sensitivity to UV light parallels the photoresponse of ZnO. The choice of ZnO is critical due to its controllable oxygen vacancies, which provide the physical basis for a strong PPC effect.^7,27–31^ This optically induced, nonvolatile conductivity is the key mechanism for replicating the memory functions demonstrated throughout this work.

**Figure 1:**
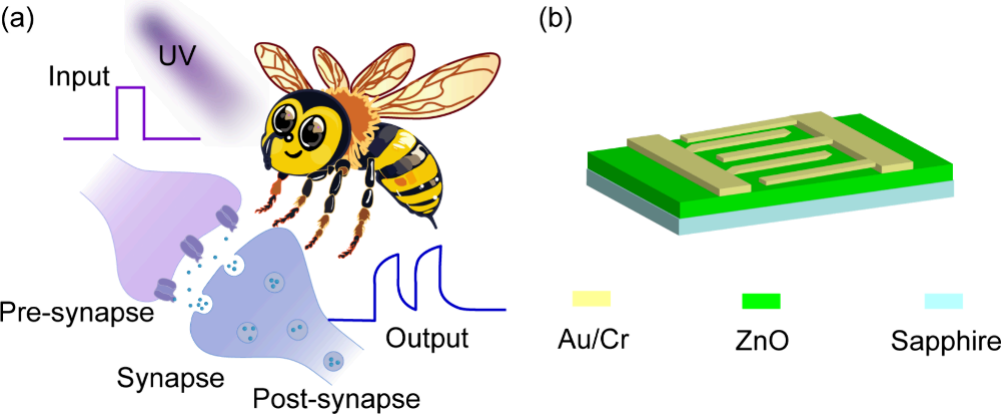
(a) Schematic representation of the biological visual system, biosynapse, and neuromodulator. (b) Structural diagram of the ZnO MSM optoelectronic device.

To investigate the optical properties of the ZnO film,^32–34^ various optical measurements were performed. Figure 2a presents the power-dependent PL spectra of the ZnO film at room temperature. The sample shows an emission peak in the UV region around 382 nm, corresponding to near band edge (NBE) excitonic recombination. With the increase of the excitation power from 0.05 to 20 mW, the intensity of the NBE increases systematically. Nevertheless, the full spectra of the ZnO thin film are dominated by a broad green luminescence (GL) band centered at around 550 nm rather than the UV luminescence, which is a well-known signature of high concentration of V_O_ defects. The intensity of this defect-related emission also increases with excitation power, which confirms that the photogenerated carriers drive both the intrinsic and defect-related emission processes. This claim is further supported by the power-dependent PL measurements at low temperature (50 K), as shown in Figure 2b. At this temperature, the intrinsic NBE emission is dramatically enhanced and dominates the spectrum, due to the suppression of nonradiative recombination pathways. The evolution of emission with temperature is plotted in Figure 2c. A key observation is the progressive decrease, or thermal quenching, of the GL band intensity with an increase in temperature. This is the characteristic of defect-related emission, where trapped carriers gain sufficient thermal energy to escape and contribute to the NBE. To quantify the carrier dynamics underlying these optical transitions, TRPL measurements were performed. The decay profiles of the emission are shown in Figure 2d. By fitting the decay curves with a biexponential function, the average carrier lifetime for each emission pathway has been determined. The NBE exhibits a very fast decay with a lifetime of 0.7 ns, while the defect-related GL shows a much longer decay lifetime of 40 ns. This slow, defect-mediated recombination process provides the essential physical basis for the long-term charge carrier retention that will be demonstrated later in the device performance. A schematic diagram of the electronic band structure of ZnO is further presented in Figure S1 for a deeper understanding of the optical properties observed above.^35–38^ The NBE corresponds to the direct and rapid recombination of free excitons across the fundamental band gap. In contrast, the prominent GL band originates from deep-level transitions involving defects. As highlighted in the schematic, the GL emission is primarily attributed to V_O_. The 2.30 eV transition, corresponding to approximately 539 nm, closely matches the experimentally observed 550 nm broad band. This GL commonly arises from the radiative recombination of an electron from the conduction band (or a shallow donor such as interstitial zinc, Zn_i_) to a deeply localized V_O_ defect level. Additionally, other V_O_ charge states, such as V_O_^2+^ (2.07 eV) and V_O_^+^ (2.45 eV), can contribute to the GL through transitions of trapped electrons to the valence band, as depicted. These defect-mediated recombination pathways, particularly those involving V_O_, directly account for the significant difference in carrier lifetimes observed in the TRPL measurements (Figure 2d). The NBE is a direct band-to-band recombination process, leading to a very short lifetime of 0.7 ns. In contrast, the GL transition involves a deeply trapped carrier. The spatial separation between the electron and the trapped hole reduces their wave function overlap, consequently lowering the recombination probability. This inefficient recombination pathway is the reason for the observed long carrier lifetime of GL emission.

**Figure 2:**
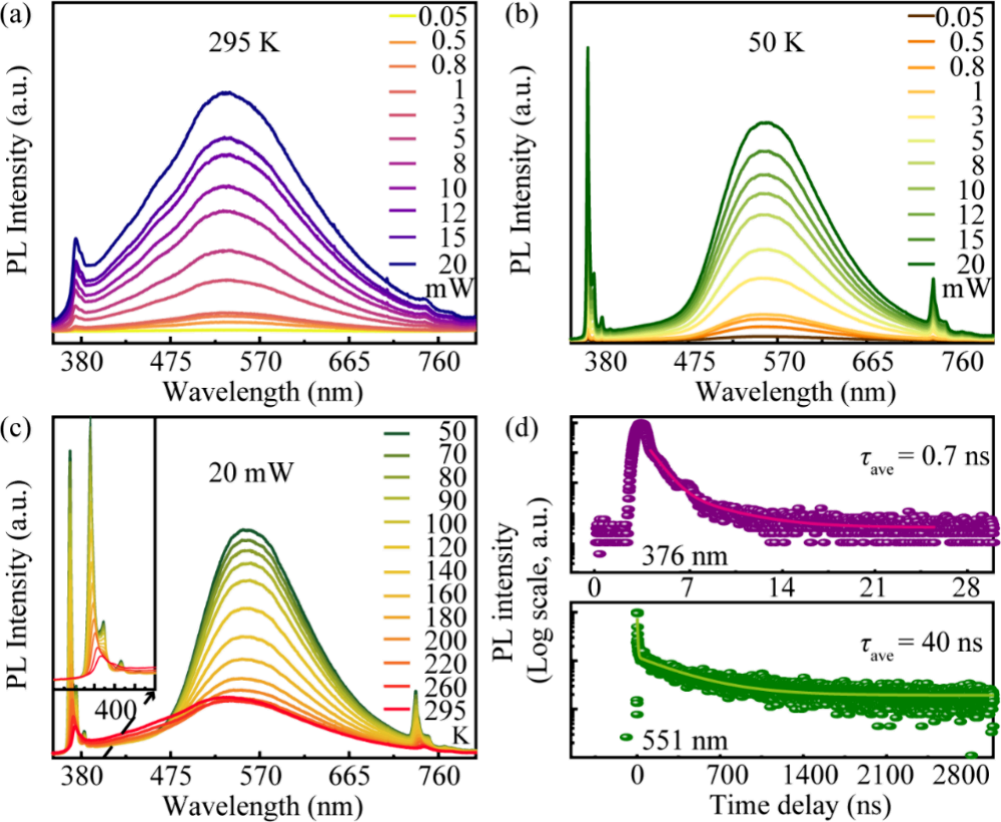
(a) Power-dependent PL spectra from the ZnO film at room temperature (295 K) with excitation power from 0.05 to 20 mW. (b) Power-dependent PL spectra of the ZnO film at low temperature (50 K) from 0.05 to 20 mW. (c) Temperature-dependent PL spectra of the ZnO film from 50 to 295 K. (d) TRPL measurements of the ZnO film at room temperature.

The structural and chemical properties of the ZnO film were characterized. The X-ray diffraction (XRD) pattern is presented in Figure S2. It shows a strong (002) peak with a broad width of around 0.29°, confirming the highly textured but structurally imperfect nature of the film. Moreover, X-ray photoelectron spectroscopy (XPS) was performed. As shown in Figure S3, the peak corresponding to oxygen vacancies accounts for approximately 43% of the O 1s signal. The results confirm that our film is a structurally imperfect and defect-rich material, providing a solid physical basis for the unique optical properties discussed below.

The fundamental optoelectronic characteristics and bioinspired synaptic functions of the ZnO devices are comprehensively investigated. The current–voltage (*I*–*V*) curves under various UV light intensities are shown in Figure 3a and Figure S4 (linear scale). The *I*–*V* curves show a linear and symmetric nature, which confirms the formation of good ohmic contact between the Au/Cr electrodes and the ZnO film. This ohmic contact provides a low-resistance path for efficient charge transport. More importantly, it ensures that the measured photoresponse is governed by the intrinsic properties of the ZnO channel rather than a rectifying Schottky barrier at the interface. As anticipated, the photocurrent increases systematically with increasing light intensity. Figure 3b shows the device’s excitatory post-synaptic current (EPSC) response to a single 5 s light pulse. The current rapidly increases upon illumination and then exhibits a slow and persistent decay after the light is removed. This characteristic nonvolatile decay is a direct manifestation of the underlying PPC effect. The PPC decay is not a single process but represents the collective release of carriers from the ensemble of V_O_ defect states. These defect states exhibit a distribution of energy levels due to local material variations. As a result, the overall photocurrent decay, *I*(*t*), is a superposition of many individual release events. This process can be well-described by a stretched-exponential function:^39^

$$I\left(t\right)=I_{0}e^{-{\left(\frac{t}{\tau }\right)}^{\beta }}$$ 1

**Figure 3:**
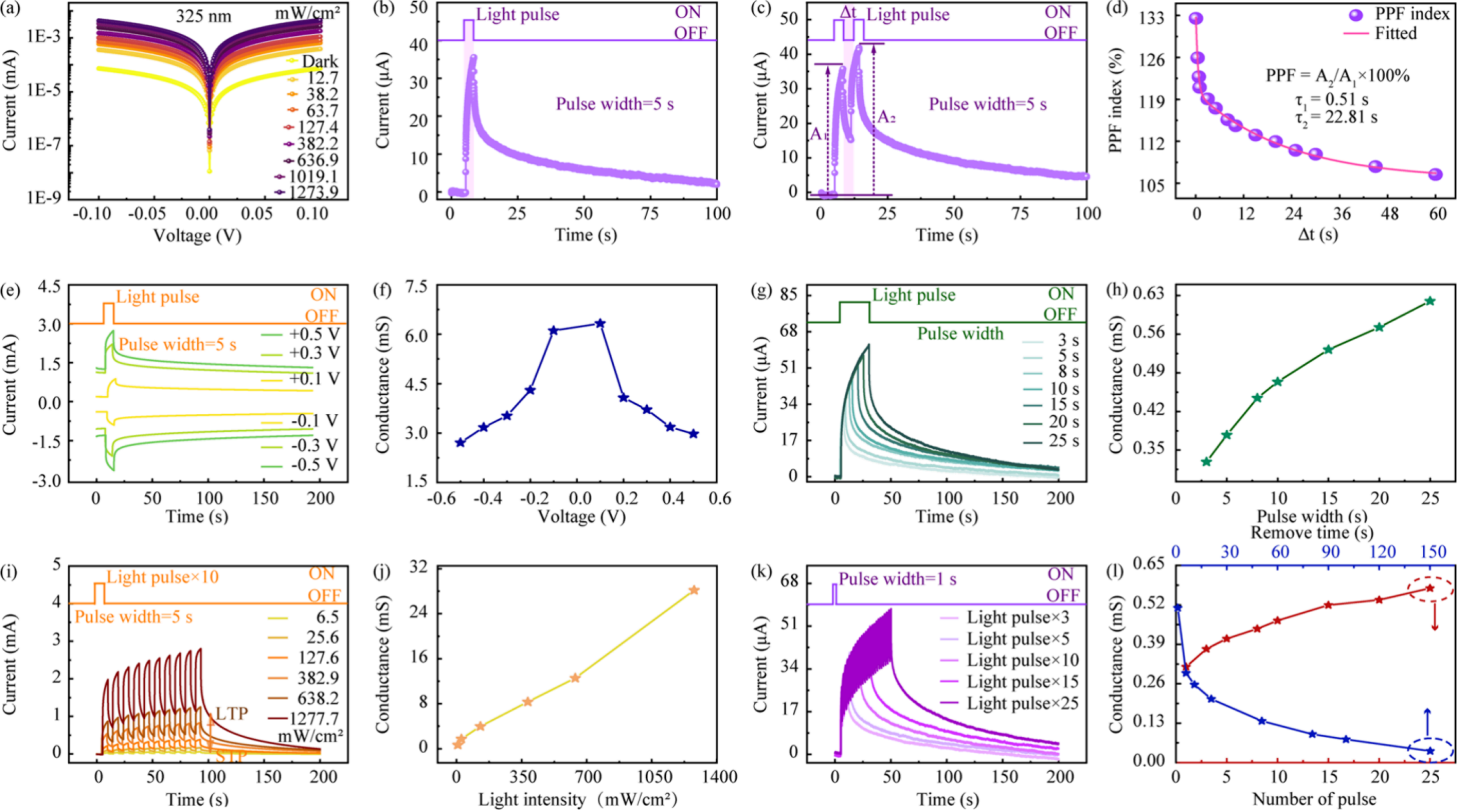
(a) *I*–*V* response characteristics of the ZnO device under varied UV light intensities. (b) EPSC triggered by a single UV light pulse. (c) Synaptic function of PPF (Δ*t* = 5 s). (d) PPF index as a function of Δ*t*. (e) EPSC as a function of reading voltage. (f) Corresponding conductance as a function of voltage. (g) EPSC dependence on pulse width. (h) Device conductance as a function of pulse width. (i) EPSC dependence on light intensity under continuous stimulations. (j) Device conductance as a function of light intensity. (k) EPSC dependence on light pulse. (l) Device conductance as a function of light pulse and remove time.

In this model, *I*_0_ represents the initial photocurrent once the light is turned off, τ is the characteristic relaxation time, and the stretching exponent β (with a value between 0 and 1) quantifies the breadth of the relaxation rate distribution. As shown in Figure S5, the plot of ln­{ln­[*I*_ppc_(0)/*I*_ppc_(*t*)]} as a function of ln­(*t*) for the PPC decay curve has been shown. An approximately linear behavior can be observed. Fitting the experimental PPC decay data from Figure 3b to this model yields an excellent fit (*R* ^2^ = 0.997) and a stretching exponent of β ≈ 0.20, as detailed in Figure S5. This fundamental PPC behavior serves as the basis for emulating STP, such as PPF, as shown in Figure 3c. When two consecutive light pulses (amplitudes A_1_ and A_2_) are applied, the second pulse elicits a larger current (A_2_ > A_1_). This is because the charge carriers generated by the first pulse have not fully relaxed before the second pulse arrives. The PPF index, defined as (A_2_/A_1_) × 100%, is plotted against the interpulse interval (Δ*t*) in Figure 3d. The index decays as Δ*t* increases, closely mimicking the behavior of the biological synapses. Fitting the decay curve with a biexponential function provides an excellent empirical model for the data, revealing two characteristic time constants. The fast component of τ_1_ = 0.51 s can be attributed to quicker relaxation pathways, such as the release of carriers from shallower trap states. The slow component of τ_2_ = 22.81 s, however, dominates long-term memory and is of primary interest. This biexponential behavior is a simplified representation of the underlying PPC effect, which arises from a continuous distribution of trap states, as described by the stretched-exponential model. The complex PPC and PPF phenomena share the same fundamental physical mechanism. The small β quantitatively confirms that the PPC arises from a broad distribution of relaxation rates, corresponding to a vast ensemble of V_O_ defect states. The slow component of PPF decay (τ_2_) is a direct probe of this same collective release process. This entire physical picture is underpinned by the deep-level nature of the oxygen vacancies, a property for which the long 40 ns carrier lifetime measured by TRPL provides definitive optical evidence. A deeper trap possesses a higher activation energy (*E*_a_) barrier for thermal release. This is the ultimate physical reason for the slow, multisecond relaxation times observed in the electrical measurements. Thus, the macroscopic synaptic memory is a direct functional consequence of the microscopic carrier dynamics within controlling V_O_ defects. An effective artificial synapse must be capable of a tunable synaptic weight and efficient operation. Figure 3e demonstrates that the magnitude of the EPSC can be precisely controlled by the applied bias voltage. The device maintains a significant and stable photoresponse across a wide range of voltages, from −0.5 to +0.5 V. The effect of this bias voltage on the synaptic weight is further quantified in Figure 3f. This figure plots the peak conductance as a function of applied voltage. The conductance exhibits a nearly symmetrical and quasilinear dependence on the magnitude of the bias voltage, further verifying the good ohmic contact of the device. This confirms that the synaptic weight of the device can be effectively and predictably modulated by the electrical bias in addition to optical stimuli. To investigate whether a self-driven photovoltaic effect exists, the photoresponse was measured at very low and zero bias, as presented in Figure S6. While a clear photoresponse is maintained at biases as low as 0.1 mV, it completely vanishes at 0 V, where the response is identical to that of the dark state. This indicates the absence of built-in potential combined with the symmetric *I*–*V* curves that pass through the origin, as shown in Figure 3a. Therefore, all observed synaptic functions are driven solely by the photoconductive effect under an external field, which validates the attribution of the PPC mechanism to the intrinsic carrier dynamics of the ZnO channel. Figure 3g shows the EPSC response of the device to a single UV light pulse of varying durations, ranging from 3 to 25 s. It can be seen that the peak photocurrent rises with the increase in pulse width. This relationship is further quantified in Figure 3h, where the peak conductance is plotted as a function of pulse width. The conductance systematically increases with longer pulse duration. Crucially, a longer pulse width not only increases the peak current but also prolongs the subsequent PPC decay time, demonstrating a form of temporal summation. The device integrates the total number of incoming photons over time, and longer exposure results in a greater accumulation of charge carriers trapped in the V_O_ defect states. This leads to a higher sustained electron concentration and thus a stronger synaptic weight. In a parallel experiment, the device’s response to varying optical intensities was characterized. Figure 3i shows the EPSC response to a train of 10 pulses at different light intensities, ranging from 6.5 to 1277.7 mW/cm^2^. The results reveal that a higher light intensity significantly enhances the potentiation of the current. This trend is quantified in Figure 3j, which plots the peak conductance against light intensity. The relationship shows a clear and strong positive correlation, indicating that the synaptic weight is modulated proportionally by the stimulus strength. Similar to the effect of pulse width, a more intensive light pulse generates a higher density of electron–hole pairs per unit of time. This leads to more efficient hole trapping by oxygen vacancies, which results in both stronger potentiation and more persistent memory with slower PPC decay. The ability of a synapse to form long-term memory through learning is critical, and this is demonstrated by the transition from STP to LTP upon repeated stimulation. This effect is explicitly quantified in Figure 3k, where increasing the number of pulses (from 3 to 25) leads to a progressively higher peak EPSC. More importantly, this repeated stimulation strengthens the persistence of memory. The key finding is shown in Figure 3l. As the number of pulses increases (on the *x*-axis), two things happen simultaneously. First, the final conductance state after the stimulus is removed becomes higher (red curve), indicating a stronger memory. Second, the current decay becomes significantly slower (blue curve), providing direct quantitative evidence that the PPC decay time is being prolonged with training. This suggests that repeated training leads to a more stable accumulation of charge carriers in the deep-level V_O_ defect states, effectively strengthening the synaptic connection. This also demonstrates the fundamental principle of the memory transition. With few pulses, the PPC decay is relatively fast, and the memory is STP. With the increase of pulse number, the PPC decay time is systematically extended. When the PPC decay time becomes significantly longer than the Δ*t*, charge carriers accumulate in a more stable configuration within the deep defect states. This prevents the synapse from fully relaxing between stimuli, leading to the formation of a nonvolatile LTP. This successful emulation of the STP-to-LTP transition is a hallmark of higher-order learning, showcasing the device’s potential for implementing complex, brain-inspired computational tasks. Further confirmation of the time-dependent potentiation is provided by the device’s frequency-dependent response, which is detailed in Figure S7. Figure S8 presents the results of in situ Kelvin probe force microscopy (KPFM). The AFM topography in Figure S8a shows the polycrystalline granular surface of the ZnO channel. The surface potential maps were then measured under the same bias conditions, both in the dark (Figure S8b) and under blue light illumination (Figure S8c). The line profiles extracted from the maps, shown in Figure S8d, quantitatively confirm a significant increase in the average surface potential from 3.05 to 3.25 V. This potential increase is direct evidence that the surface becomes more positively charged when illuminated. This finding is in perfect agreement with our proposed model, where light activates the deep-level V_O_ states and triggers photodesorption of the surface oxygen.

To elucidate the physical origins of the observed synaptic behavior, a detailed mechanistic model is proposed. The fundamental operation of any optoelectronic device can be described by three sequential processes, as illustrated in the general schematic of Figure 4a: (I) generation of electron–hole pairs by incident photons, (II) separation and drift of these carriers under an applied electric field, and (III) collection of carriers by electrodes.^40^ For our specific ZnO device, the key to its memory function lies in how these processes are modulated by intrinsic defects. Figure 4b shows the device in the ″ON″ state under UV illumination. The photons generate electron–hole pairs, and the applied bias sweeps them toward the electrodes, resulting in an increase of current. Crucially, the presence of V_O_ serves as trapping centers, which significantly alter the carrier dynamics. When the light is removed (Figure 4c), the device enters the ″OFF″ state. Instead of recombining immediately, a substantial number of photogenerated carriers remain trapped at defect levels. These trapped carriers are then slowly released as the stimulation is stopped, continuously contributing to the conductance of the device. The slow supply of released carriers sustains the current for an extended period, which is known as the PPC effect. The following sections provide a detailed explanation of the microscopic origins of this crucial persistence. The rapid increase in conductivity upon illumination is driven by two parallel and synergistic mechanisms.^41,42^ The first is a bulk defect process, as detailed in Figure 4d. Photogenerated holes are efficiently captured by neutral oxygen vacancies (V_O_^0^) in the ZnO lattice, converting them into a stable, doubly ionized state (V_O_^0^ + 2h^+^ → V_O_^2+^).^41^ This spatial separation of charges suppresses direct electron–hole recombination, leaving a substantial number of long-lived free electrons in the conduction band. The second is a surface-mediated process, as shown in Figure 4e. In the dark, adsorbed atmospheric oxygen molecules capture free electrons from the ZnO, creating a low-conductivity depletion layer (O^2^ + e^–^ → O_2_^–^).^42^ Upon UV illumination, photogenerated holes migrate to the surface, neutralizing these ions and triggering oxygen desorption (h^+^ + O_2_^–^ → O_2_). This process releases the previously trapped electrons back into the channel. The combined effects of bulk hole trapping and surface electron release lead to a significant and rapid increase in the EPSC. However, to identify the dominant mechanism responsible for the crucial PPC that enables long-term memory, the device’s photoresponse was measured in a high vacuum. As shown in Figure S9a, both the dark and light currents are significantly higher in a vacuum compared to those in ambient air. This confirms that adsorbed surface oxygen acts as an electron trap, suppressing the overall channel conductivity. The time-dependent response in Figure S9b provides definitive proof. In stark contrast to the behavior expected from a purely surface-driven device, our synapse exhibits a much stronger and more persistent PPC effect in vacuum than in air. This result is unambiguous experimental evidence that the internal, bulk oxygen vacancies are the dominant mechanism responsible for the long-term memory. The presence of environmental oxygen primarily serves to suppress the overall current and provide a faster relaxation pathway, thus modulating the intrinsic memory effect. The V_O_-dominated persistence is rooted in the slow kinetics of carrier recapture. The V_O_^2+^ states are highly stable due to a large energy barrier created by lattice relaxation, which impedes electron recombination. This physical picture aligns perfectly with the long carrier lifetime (40 ns) measured by TRPL, which is another manifestation of the same deep energy traps. Therefore, the robust PPC effect driven by V_O_ provides the solid physical foundation for all of the synaptic plasticity phenomena observed in this study. A comparison of PPC mechanisms of different devices is presented in Table S1. It shows that hole trapping by oxygen vacancies is indeed a foundational mechanism in many *n*-type oxide semiconductors such as TiO_2_ and Ga_2_O_3_.^24,26,42^ However, in 2D materials like MoS_2_, charge trapping at interfaces or in the substrate can dominate.^43^ In more complex memristive devices, ion migration is often the key process. Therefore, the research work herein provides a cross-scale understanding of the intrinsic defect mechanisms central to single-layer oxide-based synapses, offering a valuable framework for their rational design.

**Figure 4:**
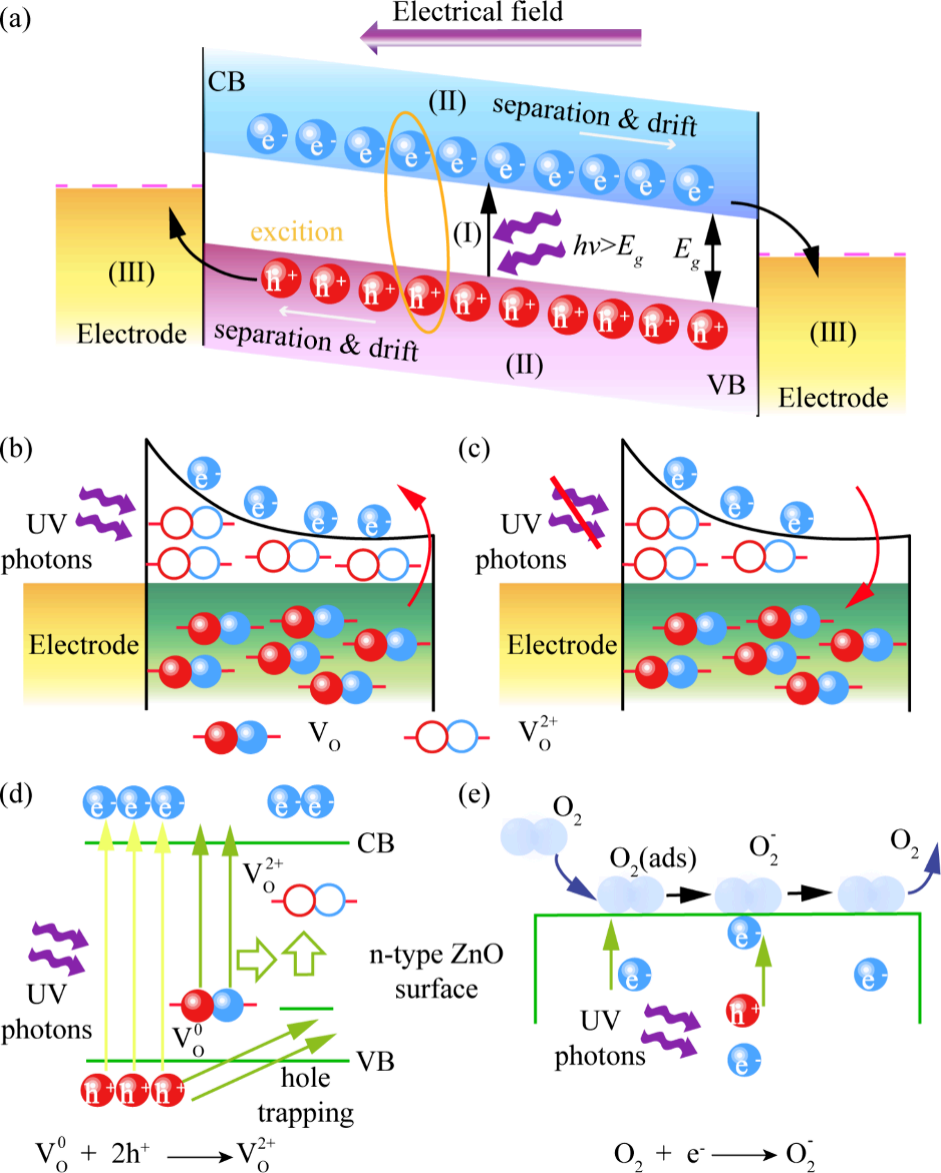
(a) Schematic illustration of the mechanism of the Au/Cr/ZnO device. An electric field is induced either by the built-in potential at the junction or by an external bias supply. (b) Band diagram of Au/Cr/ZnO interface under UV light illumination. (c) Band diagram of Au/Cr/ZnO interface with no UV light illumination. Mechanisms for PPC in ZnO films: (d) electron–hole pair excitation and V_O_ trapping of holes under UV irradiation. (e) Surface adsorption of molecular oxygen and the formation of O_2_^–^ through electron capture. Under UV illumination, photogenerated holes are trapped by the O_2_^–^ ions, resulting in the release of the O_2_ molecules into the atmosphere.

To visually demonstrate the device’s synaptic plasticity, the learning and forgetting processes were mapped onto an image of a bee, as shown in Figure 5a. During the ″learning process″, the image was exposed to a series of 5 s light pulses. After a single pulse (*N* = 1), the bee was barely visible. As the number of pulses increased to 25 (*N* = 25), the image progressively brightened and became clearer, effectively demonstrating the potentiation of synaptic weight through repeated stimulation. In contrast, during the “forgetting process” after the stimulation was removed, the bright image gradually faded over 150 s. This intuitive demonstration effectively confirms the device’s ability to emulate experience-dependent learning and memory decay, similar to biological systems. Beyond this conceptual demonstration, the device’s computational capability was rigorously tested using a standard machine learning task: handwritten digit recognition from the Modified National Institute of Standards and Technology (MNIST) data set. The multilevel conductance states of the synapse, which correspond to different levels of potentiation and decay,^44^ were used as the synaptic weights in a software-based Artificial Neural Network (ANN), as shown in Figure 5b. The network was trained and tested on the MNIST data set to recognize the digit ″7″.^45,46^ Figure 5c plots the recognition accuracy as a function of the training process. The recognition accuracy improves rapidly, reaching 90.8% after 25 epochs. During the forgetting phase (shown in the right panel), accuracy gradually declines but remains significant, stabilizing at 24.4%, even after 150 s. This result quantitatively demonstrates that the device’s analog memory states are distinct and programmable, capable of performing complex computational tasks with high accuracy. To showcase the potential for scalable, parallel hardware implementation, a 3 × 3 array of synaptic devices was simulated, as illustrated in Figure 5d. An optical pattern of digit ″7″ was projected onto this array. Figure 5e shows the conductance map of the array at various stages. After 2 learning pulses, the pattern was weak. As the number of pulses increased to 25, the conductance of the illuminated synapses strengthened significantly, making the ″7″ pattern much clearer. After the stimulus was removed, the memory of the pattern was retained, gradually fading but remaining clearly recognizable even after 150 s. The quantitative data supporting this visual simulation, which detail the specific current values for each device throughout the entire learning and forgetting process, were provided in Figures S10 and S11. This simulation confirmed that an array of these ZnO synapses could function as an artificial retina, capable of parallel image sensing, learning-based weight potentiation, and nonvolatile memory retention, opening promising avenues for advanced, integrated bionic vision systems.

**Figure 5:**
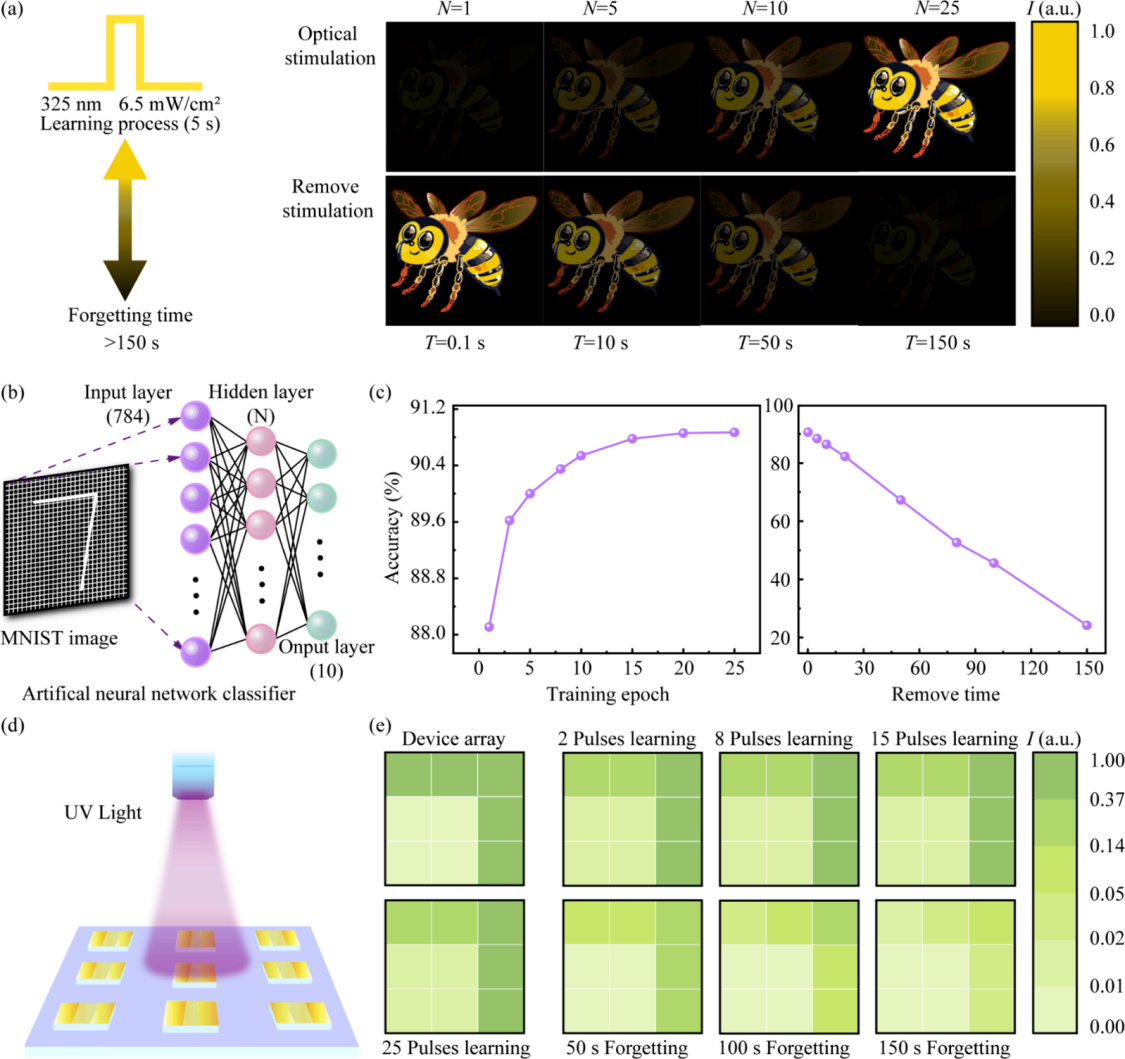
(a) Visualization of the learning and forgetting processes through EPSC mapping pattern based on a ZnO film of the optoelectronic synaptic device (325 nm, pulse width of 5 s, 6.5 mW/cm^2^). (b) Schematic of a three-layer artificial neural network. (c) Recognition accuracy for MNIST images. (d, e) Simulation of visual learning and memory function using a 3 × 3 pixel optoelectronic synaptic array with varying pulse numbers.

## Conclusions

In this work, a direct and quantitative link between atomic-scale defect dynamics and system-level neuromorphic plasticity in ZnO optoelectronic synapses was established. By revealing that nanosecond-scale carrier lifetimes in V_O_ defect states are the fundamental origin of second-scale PPC, we present a defect-level mechanism that governs both STP and LTP. This cross-time scale framework redefines oxygen vacancies, transforming them from passive imperfections into tunable memory elements. As a result, synaptic weights can be controlled predictably, eliminating the need for trial-and-error optimization. The optimized ZnO synapse operates at an ultralow bias (0.1 V), exhibits robust retention, and achieves 90.8% accuracy in handwritten digit recognition, underscoring its potential for energy-efficient artificial vision hardware. More broadly, this study offers a rational design strategy for defect-engineered neuromorphic devices, paving the way for future scalable, low power consumption, and bioinspired computing architectures.

## Methods

### Material Preparation

The ZnO thin film was grown on a *c*-sapphire substrate by the plasma-assisted molecular beam epitaxy (MBE) method. Initially, the sapphire substrate was rigorously cleaned by sequential ultrasonic bathing in acetone, isopropanol, and deionized water for 10 min each, followed by drying with a nitrogen (N_2_) stream. High-purity oxygen (6N) was employed to generate active radicals using a radio frequency (RF) plasma module. High-purity Zn (6N) source was loaded into a Knudsen cell for thermal evaporating a metal stream. During the growth, the temperature of Zn was set as 340 °C, the temperature of the substrate was steadied at 400 °C, and the flow rate of Oxygen was set as 100 sccm. The deposition time of the ZnO thin film was 2 h, and the thickness of the film layer was around 200 nm. After the growth, the ZnO thin film was annealed at 750 °C for 20 min under vacuum conditions to enhance the concentration of V_O_.

### Device Fabrication

A two-terminal ZnO photoelectric synapse with a metal–semiconductor-metal (MSM) structure was fabricated on a *c*-plane sapphire (Al_2_O_3_) substrate. An interdigitated electrode (IDE) array, which defines the core of the MSM structure, was fabricated on the ZnO surface. This was achieved by depositing a 50 nm thick Au/Cr (45 nm/5 nm) bilayer through a shadow mask via an electron beam evaporation technique. Each electrode had dimensions of 2 mm × 1 mm. The IDE design featured a finger width of 40 μm, a finger length of 1 mm, and an interelectrode spacing (channel length) of 40 μm.

### Materials Characterization

TRPL spectroscopy was performed by using a 325 nm femtosecond pulsed laser as the excitation source. Steady-state PL measurements were carried out by using a 325 nm He–Cd laser to investigate the defect states and carrier dynamics. For low-temperature power-dependent PL measurements, the temperature was fixed at 50 K, and the excitation power varied from 0.05 to 20 mW. Temperature-dependent PL measurements were conducted from 50 to 295 K within a closed-cycle helium cryostat.

### Optoelectronic Synapse Characterization

All electrical and optoelectronic measurements of the synaptic devices were conducted at room temperature in an electromagnetically shielded probe station. A Keithley 2461 SourceMeter Unit (SMU) connected to two tungsten probes was used to apply the voltage and measure current. A 325 nm He–Cd laser served as the optical stimulus source, delivering light pulses with precisely controlled intensity (6.5 to 1277 mW/cm^2^), duration (1 to 25 s), and frequency. Unless otherwise specified, a constant bias voltage of +0.1 V was applied across the device during all synaptic function tests.

### ANN Simulation

To evaluate the computational capability of the device, an ANN was constructed using Python with the PyTorch library. The network was designed for handwritten digit recognition using the MNIST data set. It consisted of a 784 neuron input layer, a 128 neuron hidden layer with a ReLU activation function, and a 10 neuron output layer with a Softmax activation function. The network was trained for 25 epochs using the Adam optimizer and a Cross-Entropy Loss function. A batch size of 32 was used during the training. The measured multilevel conductance values from the device’s LTP and long-term depression characteristics were used as the synaptic weights to train and test the network. The recognition accuracy was calculated as the percentage of correctly identified images from the 10,000-image test set. To model the memory decay (″forgetting″) process, the input pixel intensities of the images in the test set were scaled down according to the experimentally measured PPC decay curve. This approach provides a physically grounded simulation that directly maps the gradual decay of the synaptic conductance to a weakening of the input signal processed by the network.

## Supplementary Material



## Data Availability

The authors declare that the data supporting the findings of this study are available within the paper. The data that supports the findings of this study are available from the corresponding authors upon reasonable request.

## Notes

Figure S1: Diagram of the calculated defect levels in ZnO film; Figure S2: XRD pattern of ZnO film on a sapphire substrate; Figure S3: XPS result of the ZnO film; Figure S4: *I*–*V* curves demonstrating ohmic contact under different UV power densities (linear scale); Figure S5: Plot showing ln­{ln­[*I*_PPC_(0)/*I*_PPC_(*t*)]} as a function of ln­(*t*); Figure S6: Current response of ZnO optoelectronic synaptic devices under continuous excitation with light pulses of different voltages and different optical powers (0 and 127.4 mW/cm^2^); Figure S7: Current response of ZnO optoelectronic synaptic devices under continuous excitation with light pulses of different frequencies; Figure S8: Short-period microscopic visualization of light-induced surface potential dynamics via in situ KPFM; Figure S9: Short-period *I*–*t* characteristics of a ZnO device under atmospheric conditions (air) and vacuum conditions; Table S1: PPC effect in various materials; Figure S10: Response current EPSC at 25 optical pulses for each device forming the ″7″ pattern; Figure S11: Current value of each device constituting the ″7″ pattern for the corresponding number of light pulse stimulation and forgetting time conditions (PDF)
