# Supplementary material for: Defect-Driven Neuromorphic Plasticity in Planar ZnO Optoelectronic Synapses
Source: ACS Appl Mater Interfaces. 2026 Jan 13;18(3):6191–9. doi: 10.1021/acsami.5c21253 (PMC12862753; doi:10.1021/acsami.5c21253)
Supplement: Supplementary file 1 [file am5c21253_si_001.pdf]

*Supporting Information*

# Defect-Driven Neuromorphic Plasticity in Planar ZnO Optoelectronic Synapses

Zhiyuan Ren[1], Shan Wang[1], Bingheng Meng[1,2], Huan Liu[1], Qing An[1], Longxing Su[2*] and Rui Chen[3*]

[1] Department of Electrical and Electronic Engineering, Southern University of Science and Technology, Shenzhen, Guangdong 518055, P. R. China

[2] International School of Microelectronics, Dongguan University of Technology, Dongguan, Guangdong 523808, P. R. China

[3] Institute of Applied Physics and Materials Engineering, University of Macau, Avenida da Universidade, Taipa, Macao 999078, P. R. China

[*]Authors to whom correspondence should be addressed: sulongxing@dgut.edu.cn and rchen@um.edu.mo

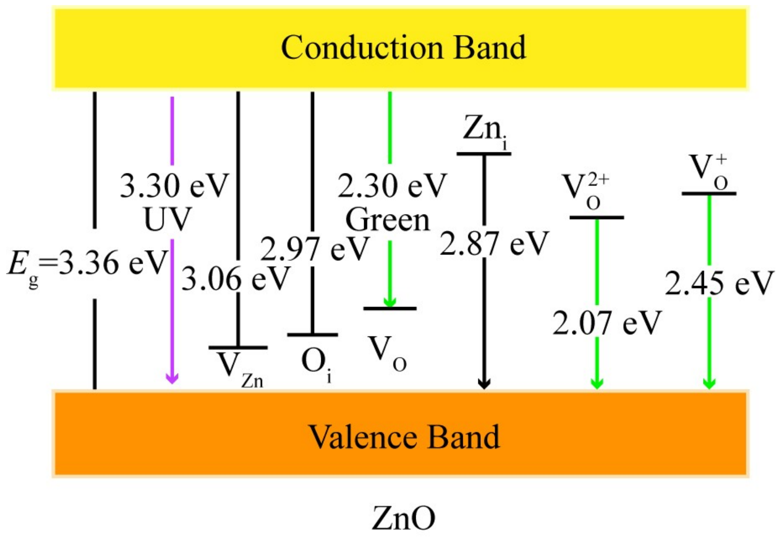


**Figure S1**. Diagram of the calculated defect levels in ZnO film.[1–4]

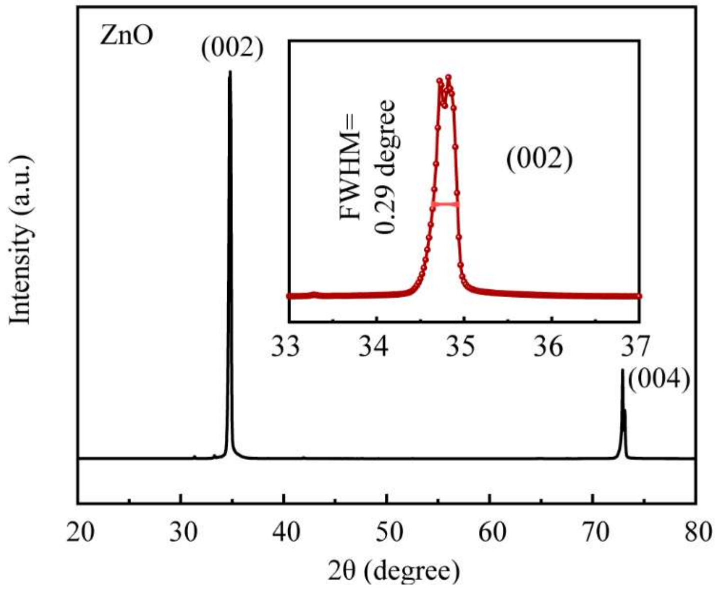


**Figure S2**. XRD pattern of ZnO film on a sapphire substrate.

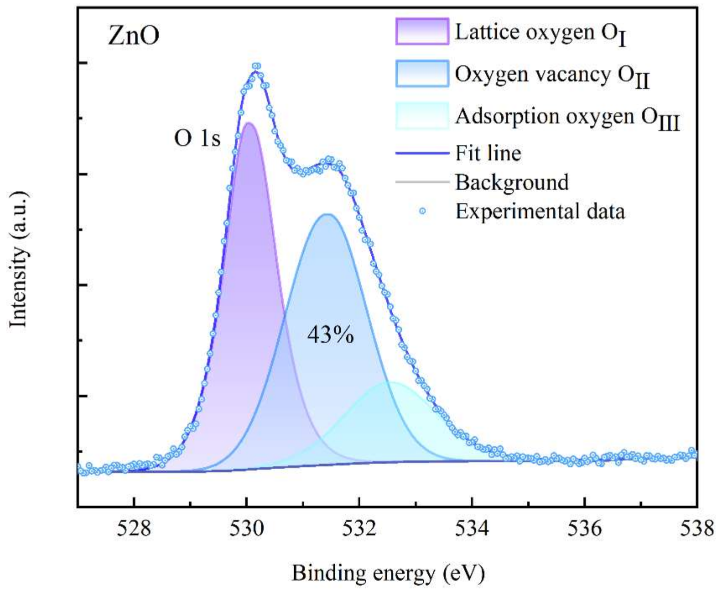


**Figure S3**. XPS result of the ZnO film.

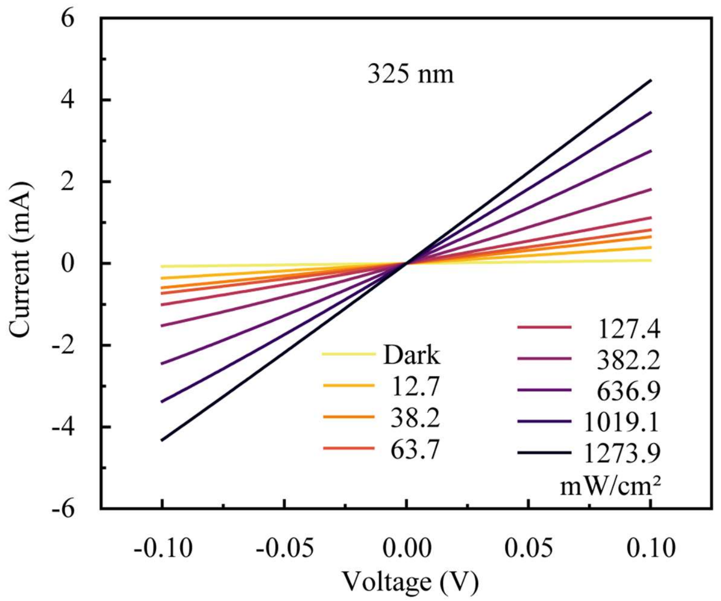


**Figure S4**. *I-V* curves demonstrating ohmic contact under different UV power density (linear scale).

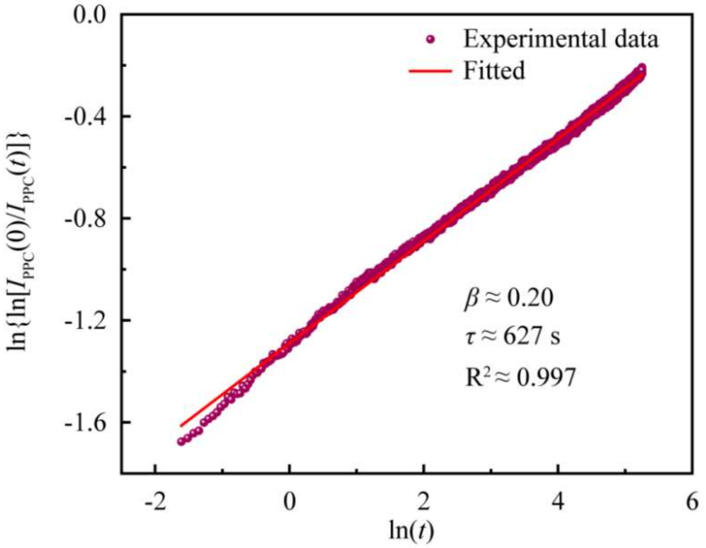


**Figure S5**. Plot showing $\ln\{\ln[I_{PPC}(0)/I_{PPC}(t)]\}$ as a function of $\ln(t)$. The approximately linear behavior confirms the long-term memory relaxation described by the stretched-exponential function.

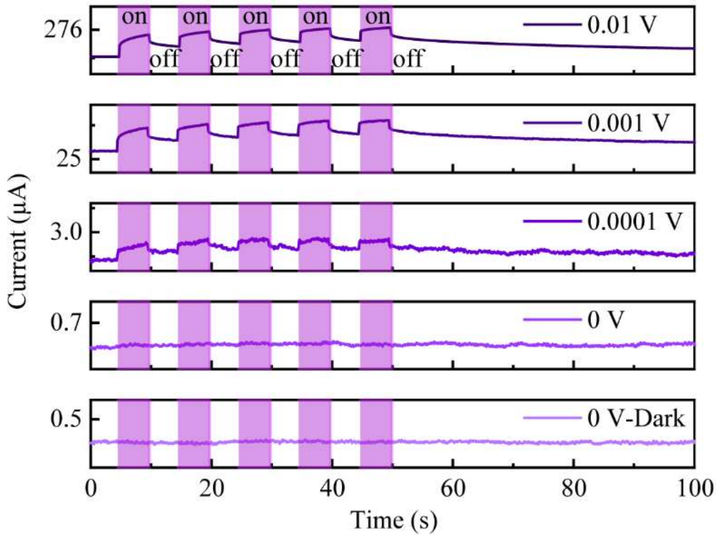


**Figure S6**. Current response of ZnO optoelectronic synaptic devices under continuous excitation with light pulses of different voltages and different optical powers (0 and 127.4 mW/cm$^2$).

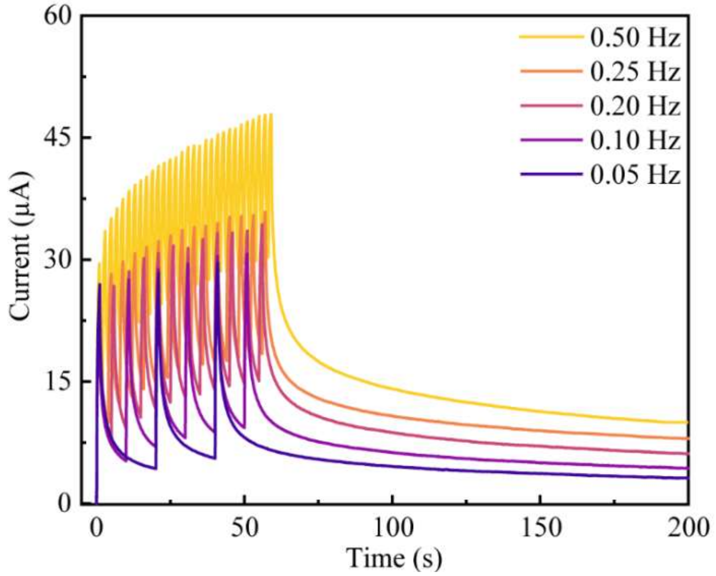


**Figure S7**. Current response of ZnO optoelectronic synaptic devices under continuous excitation with light pulses of different frequencies.

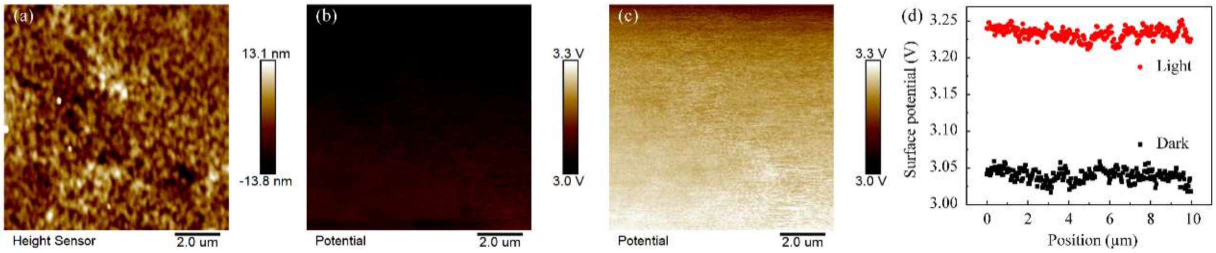


**Figure S8**. Short-period microscopic visualization of light-induced surface potential dynamics via in-situ KPFM. (a) AFM topography map of the 10×10 $\mu m^2$ ZnO channel area, showing a polycrystalline granular surface. (b) Surface potential map of the area under dark conditions with a +0.5 V bias applied. (c) Surface potential map of the area under illumination by a blue laser (~405 nm) with a +0.5 V bias applied. (d) Corresponding surface potential line profiles extracted horizontally across the center of the channel from maps (b) and (c), quantitatively confirming the average potential increase from ~3.05 V in the dark to ~3.25 V under light illumination.

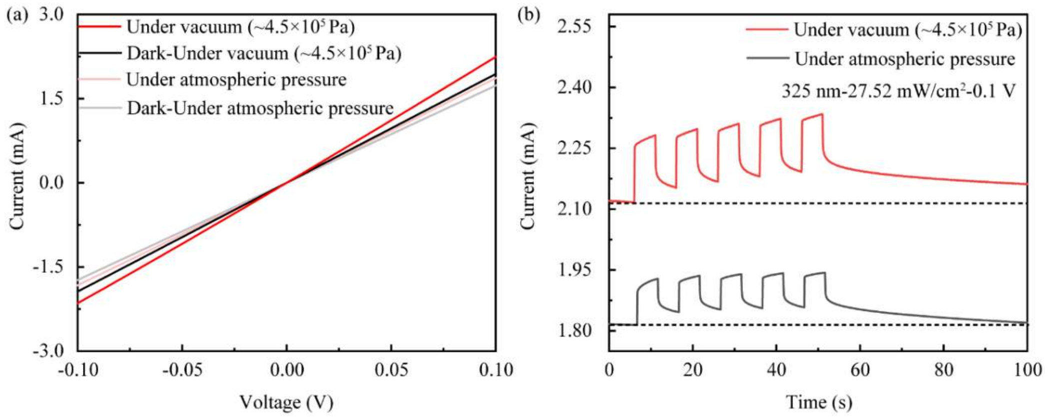


**Figure S9**. Short-period (a) I-t and (b) I-V characteristics of a ZnO device under atmospheric conditions (air) and vacuum conditions.

**Table S1.** PPC effect in various materials

| Material | PPC Mechanism | Read Voltage (V) | E [event-1] /Operation energy | Terminals | References |
|---|---|---|---|---|---|
| $TiO_2$ | Holes left in the valence band | -1 | 7.6 $mW/cm^2$ | Two | [5] |
| $VO_2$ | Photothermal processes | 7 | 230 $W/cm^2$ | Two | [6] |
| $Ga_2O_3$/Au | Schottky barrier | 20 | 446 $\mu W/cm^2$ | Two | [7] |
| AlGaN/GaN | 2DEG with photocarrier trapping | $V_{GS}$=-3 $V_{DS}$=10 | 5 $\mu W/cm^2$ | Three | [8] |
| GaN/$Ga_2O_3$/GaN | Voltage-programmed oxygen vacancy | 5 | ~584 pJ | Two | [9] |
| $SnO_2$ NWs | Surface state | $V_{GS}$=-3 $V_{DS}$=1 | ~215.6 pJ | Three | [10] |
| $MoS_2$ | Charge trapping | $V_{GS}$=-0.5 $V_{DS}$=0.1 | / | Three | [11] |
| ZnO NWs | Surface oxygen | $V_{GS}$=0 $V_{DS}$=1 | ~1 pJ | Three | [12] |
| $In_2O_3$/ZnO | Interface charge trapping | 0.6 | ~200 pJ | Two | [13] |
| $O_D$/IGZO/$O_R$ | Oxygen vacancy charge state | 0.01 | 20 $\mu W/cm^2$ | Two | [14] |
| Au/ZnO/Pt | Oxygen vacancy charge state | 0.01 | 36 $\mu W/cm^2$ | Two | [15] |
| ZnO | Oxygen vacancy defects | 0.1 | ~80 pJ | Two | This work |

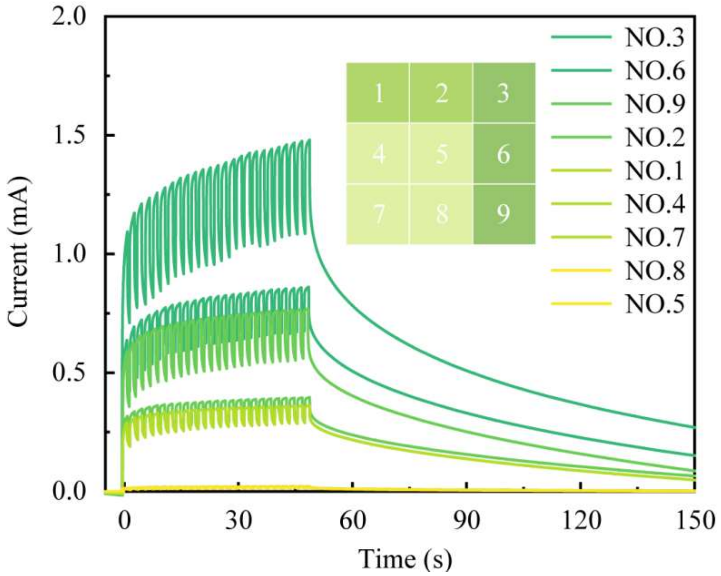


**Figure S10**. Response current EPSC at 25 optical pulses for each device forming the "7" pattern.

| | NO. 1 | NO. 2 | NO. 3 | NO. 4 | NO. 5 | NO. 6 | NO. 7 | NO. 8 | NO. 9 |
|---|---|---|---|---|---|---|---|---|---|
| 2 pulses learning | 3.26134E-4 | 3.63106E-4 | 0.00119 | 1.67826E-5 | 1.54068E-5 | 7.17796E-4 | 1.59327E-5 | 1.58104E-5 | 6.52384E-4 |
| 8 pulses learning | 3.55793E-4 | 3.97234529E-4 | 0.001339417 | 1.95159E-5 | 1.7816E-5 | 8.16117939E-4 | 1.82502E-5 | 1.8079E-5 | 7.33235089E-4 |
| 15 pulses learning | 3.67248E-4 | 4.06716949E-4 | 0.001420496 | 2.06877E-5 | 1.89024E-5 | 8.56398289E-4 | 1.91238E-5 | 1.87917E-5 | 7.63712E-4 |
| 25 pulses learning | 3.7959185E-4 | 4.15032302E-4 | 0.001495617 | 2.14097E-5 | 1.93424E-5 | 8.64042764E-4 | 1.9748303E-5 | 1.9568937E-5 | 7.94362859E-4 |
| 50 s forgetting | 1.33562E-4 | 1.52875442E-4 | 5.18293125E-4 | 7.06868E-6 | 5.60147E-6 | 3.13532224E-4 | 6.19309E-6 | 6.05369E-6 | 2.60606659E-4 |
| 100 s forgetting | 6.65769E-5 | 1.05504E-4 | 2.97319795E-4 | 6.41434E-6 | 4.96341E-6 | 1.63597344E-4 | 5.91797E-6 | 5.2464E-6 | 1.01271E-4 |
| 150 s forgetting | 1.78326E-5 | 3.06195E-5 | 1.89113825E-4 | 5.0287E-6 | 4.56582E-6 | 7.12503E-5 | 5.2822E-6 | 5.01677E-6 | 2.93945220E-5 |

**Figure S11**. Current value of each device constituting the "7" pattern for the corresponding number of light pulse stimulation and forgetting time conditions.

## REFERENCES


[1] Galdámez-Martinez, A.; Santana, G.; Güell, F.; Martínez-Alanis, P. R.; Dutt, A. Photoluminescence of ZnO Nanowires: A Review. *Nanomaterials* **2020**, *10* (5), 857.

[2] Pengshou, X.; Yuming, S.; Chaoshu, S.; Faqiang, X.; Haibin, P. Electronic Structure of ZnO and Its Defects. *Sci. China Ser. Math.* **2001**, *44* (9), 1174–1181.

[3] Mondal, P. Effect of Oxygen Vacancy Induced Defect on the Optical Emission and Excitonic Lifetime of Intrinsic ZnO. *Opt. Mater.* **2019**, *98*, 109476.

[4] Wei, X. Q.; Man, B. Y.; Liu, M.; Xue, C. S.; Zhuang, H. Z.; Yang, C. Blue Luminescent Centers and Microstructural Evaluation by XPS and Raman in ZnO Thin Films Annealed in Vacuum, $N_2$ and $O_2$. *Phys. B Condens. Matter* **2007**, *388* (1–2), 145–152.

[5] Ben Amor, F.; Hamdaoui, N.; Mezni, A.; Ajjel, R. Effect of Au Nanoparticles in Persistent Photoconductivity and Dielectric Relaxations of Anatase $TiO_2$. *Opt. Mater.* **2023**, *138*, 113645.

[6] Lee, G. Y.; Mun, B. S.; Ju, H. Observation of Giant Persistent Photoconductivity on Vanadium Dioxide Thin Film Device. *Appl. Mater. Today* **2021**, *22*, 100894.

[7] Zhou, H.; Cong, L.; Ma, J.; Li, B.; Xu, H.; Liu, Y. Suppression of Persistent Photoconductivity in High Gain $Ga_2O_3$ Schottky Photodetectors. *Chin. Phys. B* **2021**, *30* (12), 126104.

[8] Zhang, H.; Liang, F.; Yang, L.; Gao, Z.; Liang, K.; Liu, S.; Ye, Y.; Yu, H.; Chen, W.; Kang, Y.; Sun, H. Superior AlGaN/GaN-Based Phototransistors and Arrays with Reconfigurable Triple-Mode

Functionalities Enabled by Voltage-Programmed Two-Dimensional Electron Gas for High-Quality Imaging. *Adv. Mater.* **2024**, *36* (36), 2405874.

[9] Feng, S.; Li, J.; Feng, L.; Liu, Z.; Wang, J.; Cui, C.; Zhou, O.; Deng, L.; Xu, H.; Leng, B.; Chen, X.; Jiang, X.; Liu, B.; Zhang, X. Dual-Mode Conversion of Photodetector and Neuromorphic Vision Sensor via Bias Voltage Regulation on a Single Device. *Adv. Mater.* **2023**, *35* (49), 2308090.

[10] Chen, Y.; Qiu, W.; Wang, X.; Liu, W.; Wang, J.; Dai, G.; Yuan, Y.; Gao, Y.; Sun, J. Solar-Blind $SnO_2$ Nanowire Photo-Synapses for Associative Learning and Coincidence Detection. *Nano Energy* **2019**, *62*, 393–400.

[11] Wu, Y.; Liu, C.; Chen, S.; Shih, F.; Ho, P.; Chen, C.; Liang, C.; Wang, W. Extrinsic Origin of Persistent Photoconductivity in Monolayer $MoS_2$ Field Effect Transistors. *Sci. Rep.* **2015**, *5* (1), 11472.

[12] Shen, C.; Gao, X.; Chen, C.; Ren, S.; Xu, J.; Xia, Y.; Wang, S. ZnO Nanowire Optoelectronic Synapse for Neuromorphic Computing. *Nanotechnology* **2021**, *33* (6), 065205.

[13] Kumar, M.; Abbas, S.; Kim, J. All-Oxide-Based Highly Transparent Photonic Synapse for Neuromorphic Computing. *ACS Appl. Mater. Interfaces* **2018**, *10* (40), 34370–34376.

[14] Hu, L.; Yang, J.; Wang, J.; Cheng, P.; Chua, L. O.; Zhuge, F. All-Optically Controlled Memristor for Optoelectronic Neuromorphic Computing. *Adv. Funct. Mater.* **2020**, *31* (4), 2005582.

[15] Yang, J.; Hu, L.; Shen, L.; Wang, J.; Cheng, P.; Lu, H.; Zhuge, F.; Ye, Z. Optically Driven Intelligent Computing with ZnO Memristor. *Fundam. Res.* **2024**, *4* (1), 158–166.
